# Brain and Behavior in Decision-Making

**DOI:** 10.1371/journal.pcbi.1003700

**Published:** 2014-07-03

**Authors:** Peter Cassey, Andrew Heathcote, Scott D. Brown

**Affiliations:** School of Psychology, University of Newcastle, Newcastle, New South Wales, Australia; Indiana University, United States of America

## Abstract

Speed-accuracy tradeoff (SAT) is an adaptive process balancing urgency and caution when making decisions. Computational cognitive theories, known as “evidence accumulation models”, have explained SATs via a manipulation of the amount of evidence necessary to trigger response selection. New light has been shed on these processes by single-cell recordings from monkeys who were adjusting their SAT settings. Those data have been interpreted as inconsistent with existing evidence accumulation theories, prompting the addition of new mechanisms to the models. We show that this interpretation was wrong, by demonstrating that the neural spiking data, and the behavioural data are consistent with existing evidence accumulation theories, without positing additional mechanisms. Our approach succeeds by using the neural data to provide constraints on the cognitive model. Open questions remain about the locus of the link between certain elements of the cognitive models and the neurophysiology, and about the relationship between activity in cortical neurons identified with decision-making vs. activity in downstream areas more closely linked with motor effectors.

## Author Summary

In everyday life we constantly balance urgency against caution when making decisions – known as the speed-accuracy tradeoff. Traditionally, computational cognitive theories called “evidence accumulation models” have explained the speed accuracy tradeoff as changes in the amount of evidence necessary to trigger the selection of a response. Recent work recording firing rates from the neurons of monkeys while they made decisions revealed an apparent discrepancy between the firing rates and the way evidence accumulation models explain the speed-accuracy tradeoff. This discrepancy was interpreted as showing that traditional parameter settings were wrong, and that the fundamental dynamic structure of the evidence accumulation model required an addition. This result is important because it calls into question nearly half a century of cognitive science. We show instead that only the parameter settings need be adjusted, not the basic model structure, in order to account for the behavioural data and the recorded neural data. Underlying our results was an integrated approach to the neural and behavioral data, allowing both streams to inform the theoretical development.

## Introduction

The speed-accuracy tradeoff (SAT) is an important element of day-to-day functioning for humans, managing the balance between making decisions correctly while not wasting time. This balance of caution with urgency has been studied for decades in humans (e.g., [1]) and has been observed in the behavior of many other animals, from rats to bees and even slime mould [2]–[5].

The dominant cognitive theoretical framework for explaining the SAT is “sequential sampling”, also known as “evidence accumulation”. Accumulator theories explain many aspects of decision-making behavior by assuming that decisions are made by gradually accumulating evidence from the environment in favour of each possible choice. The first choice to accumulate a threshold amount of evidence is selected. Accumulator theories most naturally explain the SAT in terms of changes in the evidence threshold. When a high threshold is set, a lot of evidence must be collected before a decision is made, leading to slow but careful decisions. Conversely, when a low threshold is set decisions are made quickly, but are more often wrong because they are based on too little evidence. Using this conventional parameterization, accumulator models have a long and successful history of providing detailed, quantitative accounts of many different aspects of decision-making, including the SAT [6]–[10]. In addition, accumulator models now underpin hundreds of applied studies, where the decision-making theory is used as a tool to understand important problems including clinical disorders [11], alcohol intoxication [12], and sleep deprivation [13].

More recently, neurophysiological research has provided insights into the neural underpinnings of decision-making. For example, Glimcher [14] reviewed a broad range of research using saccadic decision making as a way to understand the neural bases of decision making in primates, from historical beginnings, through seminal studies, to current theories. Shadlen and Kiani [15] explore the neural bases of perceptual decision making and draw links with other categories of decision making. At a more theoretical level, links have been developed between neurophysiological structures and cognitive models of decision-making [16]–[21]. According to Schall's [22] comprehensive overview, “movement” neurons in the frontal eye fields (FEF) of monkeys are identified with the process of evidence accumulation: those neurons accumulate evidence towards a threshold, and a behavioural response follows soon after. “Visually responsive” neurons in the FEF represent the evidence that is being accumulated – the strength of evidence in favour of each choice, which is sometimes called the “drift rate” [23]. There are, however, alternative accounts – see, for example, [24].

These links between neurophysiology and cognitive models allow the possibility of testing cognitive models on their ability to simultaneously account for both behavioral and neural data. Simultaneously addressing both data streams is a difficult statistical problem, and the most appropriate and coherent method for doing so is a topic of current research [25]. Recently, in comparing cognitive model predictions for behavioral and neural data, Heitz and Schall [26] identified an apparent discrepancy between neural mechanisms of the speed-accuracy tradeoff and the account given by evidence accumulation models. Heitz and Schall undertook the first neural investigation of the SAT. Two monkeys made repeated perceptual decisions, sometimes under pressure to be careful, and other times under pressure to be fast. Emphasis on accuracy vs. speed was indicated by a colored cue before each decision, and enforced by rewards and time-outs for responding too quickly or too slowly. The activity of neurons in the monkeys' FEFs was recorded during decisions.

The most striking finding reported by Heitz and Schall was a disconnect between cognitive accounts of the SAT and the neural data. When they applied an accumulator model to the behavioral data alone (response time and accuracy), the model yielded the conventional account of the SAT: emphasis on accuracy over speed was mediated by an increased evidence threshold. The neural data showed something quite different, with *decreased* thresholds for accuracy emphasis, and large changes in the speed of evidence accumulation across conditions. This evidence suggests that the standard accumulator theories – which have successfully explained perceptual decision-making data for nearly 50 years – do not map cleanly onto neural data. Building on this conclusion, Heitz and Schall developed a new accumulator model (their integrated accumulator model, or “iA”) with two changes: less strict constraint on the way parameters change between speed and accuracy emphasis conditions, and an additional, nonlinear accumulation phase added following the regular evidence accumulation process. These two assumptions allowed the iA model to capture the activity recorded from cortical neurons identified with decision-making, as well as some pre-existing knowledge about the behaviour of downstream neurons, related to response effectors. However, the new iA accumulator model has some important disadvantages over conventional models, including greater complexity, and a lack of practical and tractable estimation procedures. These disadvantages must be carefully weighed against the model's advantages, as the computational tractability of evidence accumulation models has been one of their great strengths, supporting application and aiding understanding in a wide range of applied fields.

We studied this problem by investigating whether a standard accumulator model, without any changes to the underlying process of evidence accumulation or its tractability, might be made to fit both the behavioral and neural data recorded by Heitz and Schall. This resolves the apparent disconnect between conventional accumulator models and Heitz and Schall's neural data, although it leaves open some questions about activity in brainstem neurons. Our modelling procedure corrects some problems in the procedures used by Heitz and Schall, and also uses the neural data to enforce strong constraints (quantitative constraints, where possible) on the cognitive model. This results in a model which simultaneously fits the behavioral data and is consistent with the neural data, without making new assumptions about the dynamics of the evidence accumulation process.

## Results

### How did the monkeys interpret speed vs. accuracy instructions?

**Figure 1:**
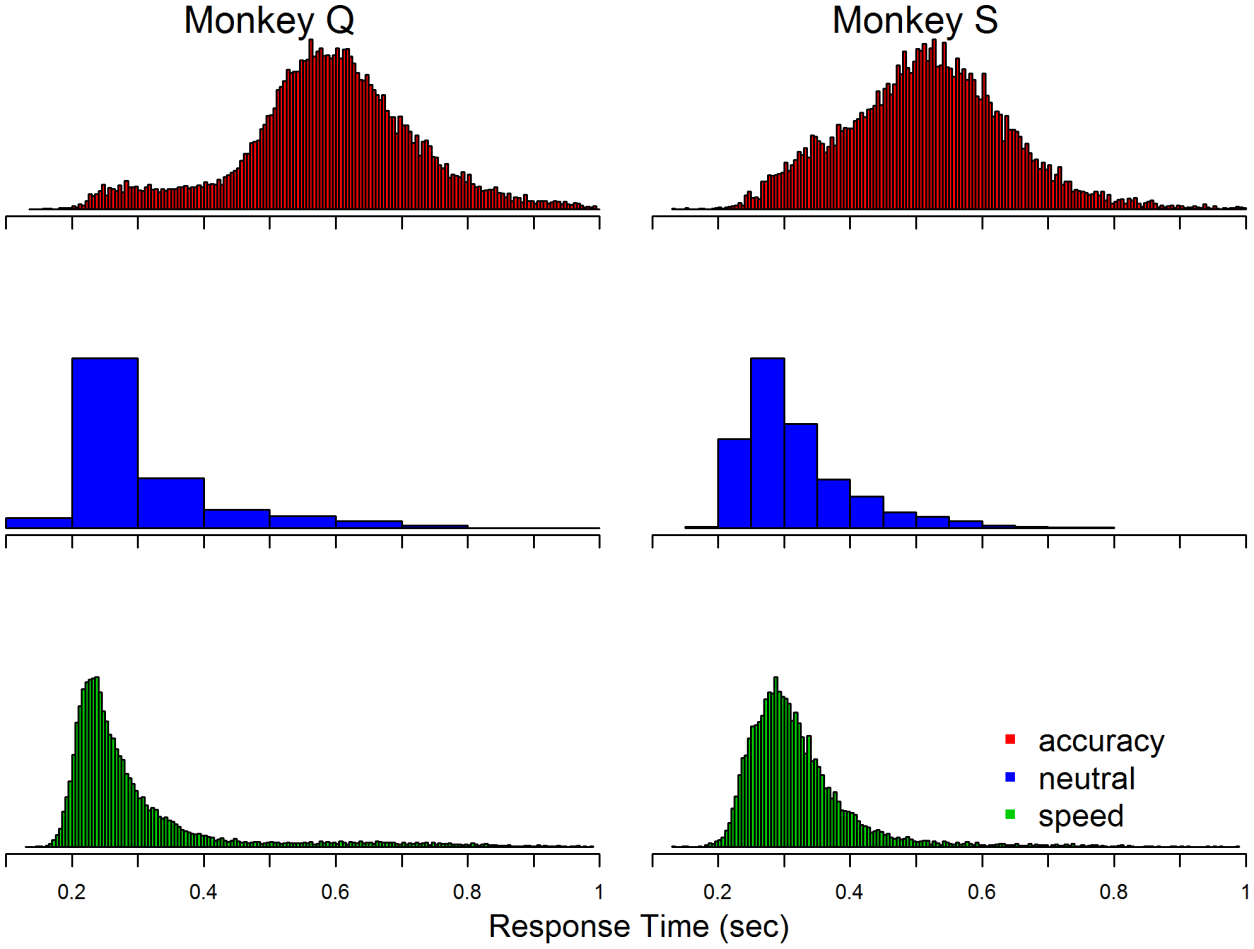
Response time histograms, plotted separately for speed, neutral and accuracy conditions, for Monkeys Q and S. The neutral-emphasis condition uses wider histogram bins because there were many fewer data from that condition. Histograms are collapsed over correct and incorrect responses, but see Figure 2 for more information.

Figure 1 shows the histograms of response times for the two monkeys, separated into data from the speed-, neutral- and accuracy-emphasis conditions. It is apparent from these data that the monkeys naturally favour speeded responding. For example, data from the neutral condition (in which monkeys could choose their own level of speed emphasis) are quite similar to data from the speed-emphasis condition, but very different to data from the accuracy-emphasis condition. Data from the speed-emphasis and neutral-emphasis conditions exhibit the typical shape for response time distributions that has been observed in thousands of studies (see [27]), with a sharply increasing left tail and a slowly decreasing right tail. Data from the accuracy-emphasis condition, in contrast, show a very unusual broadening of the left tail. The data responsible for this broadening correspond with the response time distributions observed for the speed-emphasis condition, which suggests an explanation for these unusual results: on some decisions when the monkeys were given an accuracy-emphasis cue they instead reverted to behavior more consistent with speed emphasis. Rather than trying to separate decisions for which the monkeys neglected the accuracy-emphasis cue, we directly modeled the process of cue neglect and estimated the proportion of decisions on which it occurred.

The neural recordings reported by Heitz and Schall [26] also revealed an interesting effect of the speed-accuracy tradeoff manipulation. There was more activity from visually responsive neurons under speed-emphasis than accuracy emphasis (see their Figure 2). In response to a speed-emphasis cue, visually responsive neurons increased the baseline activity exhibited in the absence of visual stimulation. They also showed increased activity in response to visual stimuli, whether that stimulus was a target or not. These effects are important because visually responsive neurons from the FEF are assumed to correspond to the evidence that is to be accumulated. If the linking assumptions that Heitz and Schall have made between accumulator models and the neurophysiology are right, the neural data suggest that the two monkeys did not adjust their speed-accuracy settings in the conventional manner. Rather, the neural data suggest that the monkeys increased their rate of evidence accumulation in the speed-emphasis condition.

**Figure 2:**
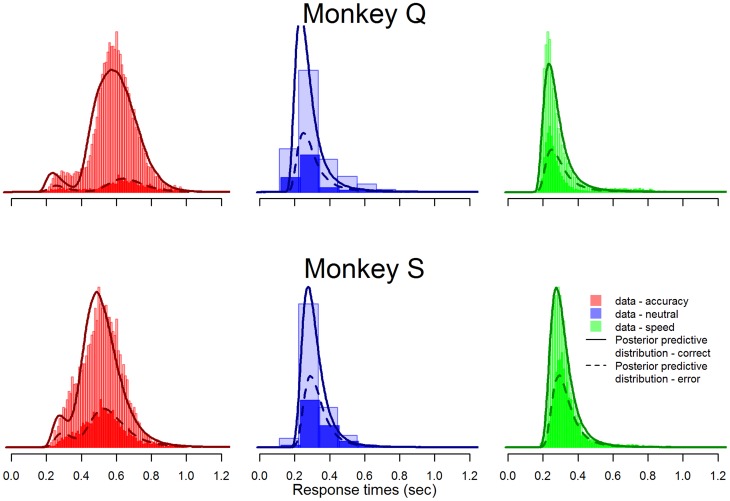
Joint probability distribution functions over response time and choice. Observed data are shown by coloured histograms. Lightly shaded histrograms represent correct responses with superimposed darker histograms representing incorrect responses. Model posterior predictive distributions are overlaid as lines.

## Models

### A conventional accumulator model accounts for the data

Following Heitz and Schall [26], we used the linear ballistic accumulator (LBA) model, which is a well-validated and extensively tested theory of simple decision-making [6]. The LBA represents a two-choice decision as a race between two accumulators, with a response triggered by whichever accumulator reaches threshold first. The model's parameters include: the threshold amount of evidence required to trigger a response (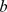); the amount of evidence or bias toward a particular response prior to evidence accumulation (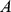); the rate of evidence accumulation, also known as “drift rate” (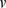); and the time taken by perceptual and motor processes (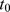). We included one extra parameter in our model, to measure the tendency for monkeys to sometimes disregard accuracy-emphasis cues. This was instantiated by modelling performance in the accuracy-emphasis condition as a mixture between the speed-emphasis and accuracy-emphasis settings, with mixing proportion denoted by 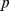. To keep things simple, we forced the model to make identical predictions for the speed-emphasis and neutral-emphasis conditions, reflecting the much smaller apparent differences between data from those two conditions vs. data from the accuracy-emphasis condition. This simplifying constraint could be relaxed if greater detail was required from the model predictions. In one final simplification, we set the start point variability parameter to a fixed small value (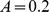).

We used the neurophysiological data recorded by Heitz and Schall to provide strong constraints on our behavioral model. Firstly, we forced the model to estimate different drift rate parameters between the speed-emphasis and accuracy-emphasis settings. This was inspired by the neural recordings reported in Heitz and Schall's Figure 2C, and the link between visually responsive neurons and drift rates. In particular, we hypothesized that speed emphasis would lead to larger mean drift rates for both racing accumulators, and also greater variance in drift rates than accuracy emphasis. Our assumption that the monkeys might have implemented a speed-accuracy tradeoff partly via changing drift rates is not just consistent with the neural data, it also has some precedence in behavioral experiments, especially when there are very large changes in performance between speed-emphasis and accuracy-emphasis conditions [28]–[31]. A second, even more precise, constraint was imposed on the behavioral model. Heitz and Schall's neural recordings exhibited a 20% increase in the firing rate threshold between speed-emphasis and accuracy-emphasis conditions. To force the cognitive model to be consistent with the neural data, we constrained the parameters for the decision threshold to have exactly this difference.

With these assumptions, our LBA model had fewer free parameters than the iA model of Heitz and Schall. Putting aside parameters used by Heitz and Schall [26] to model the neutral-emphasis condition, and by us to model cue neglect, our LBA model had seven free parameters and the iA model had nine. We used Bayesian methods to estimate the parameters of the LBA model from the data, via Markov Chain Monte Carlo integration with proposals generated by a differential evolution algorithm [32], [33]. More details of the sampling method, as well as marginal posterior distributions for all parameter estimates for both monkeys, are given in Text S1 and Figures S1 and S2.

Table 1 shows the mean of the marginal posterior distribution for each parameter. The monkeys disregarded the accuracy-emphasis cues on around 10% of trials. When they did attend to the cue, the distribution of drift rates under speed emphasis was larger and more variable (parameter 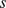) than under accuracy emphasis. This difference is consistent with both the neural data and with the parameter estimates from Heitz and Schall's iA model. Under speed emphasis mean drift rate was higher both for the accumulator representing a neuron with a target in its receptive field (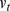) and and for the accumulator representing a neuron with a distractor in its receptive field (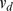).

**Table 1:** LBA parameter estimates.

*Monkey:*		*Q*		*S*	
*Condition:*		*Accuracy*	*Speed*	*Accuracy*	*Speed*
Non-decision time (t_0_)		0.10		0.09	
Mixture (*p*)		0.08		0.12	
Threshold (*b*)		0.24	0.29	0.42	0.50
*Mean Drift Rate, RF with:	Target (*v_t_*)	0.66	2.30	1.16	2.78
	Distractor (*v_d_*)	0.40	1.41	0.81	2.11
Drift Rate Std. Dev. (*s*)		0.10	(1)	0.28	(1)

LBA parameter estimates for both monkeys. Parameter 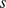 was fixed arbitrarily at 1.0 in the speed-emphasis condition, to satisfy a mathematical scaling property of the model. Parameter 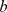 for the speed condition was fixed at a factor of 1.2 of 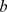 for the accuracy condition. Measurement units: 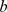 in arbitrary units of evidence; 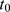 in seconds; 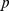 dimensionless; other parameters in evidence units per second. “RF” = receptive field.

Note that parameter 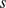 was fixed arbitrarily at 1.0 in the speed-emphasis condition, to satisfy a mathematical scaling property of the model [34]. In other applications, an alternative constraint is sometimes applied instead to satisfy the same scaling property (namely, 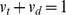). Heitz and Schall actually applied both these constraints simultaneously, which seriously impacts the model's ability to fit data. To illustrate, when we enforced the double constraint on our model, the resulting model fit was very poor indeed (see Text S2 and Figure S3). It is likely that this over-constraint similarly reduced the ability of the LBA models analysed by Heitz and Schall to fit the data adequately, which may have undermined their conclusions.

Even though our LBA model was tightly constrained by assumptions drawn from Heitz and Schall's neural data, the LBA model fit the behavioral data quite well. Figure 2 shows the goodness-of-fit displayed using probability density functions for each monkey, conditioned on correct vs. incorrect responses, and drawn separately for speed-, neutral- and accuracy-emphasis. The data are shown by histograms, and the LBA model predictions are shown by smooth lines. Model predictions were generated by generating data from the model using parameter settings sampled randomly from the posterior distribution. The model predictions align closely with the data for both monkeys, except where the model predicts, for Monkey Q, slightly too-high response accuracy for the neutral-emphasis condition and too-low response accuracy and speed- and accuracy-emphasis conditions. Most importantly, when compared with Heitz and Schall's Figure 6C, the qualitative fit of the LBA model is at least as good as that of the iA model which was developed in part to fit this data set. Quantitative comparisons of goodness-of-fit might be even more illuminating here, but are not available for the iA model due to its computational intractability.

**Figure 3:**
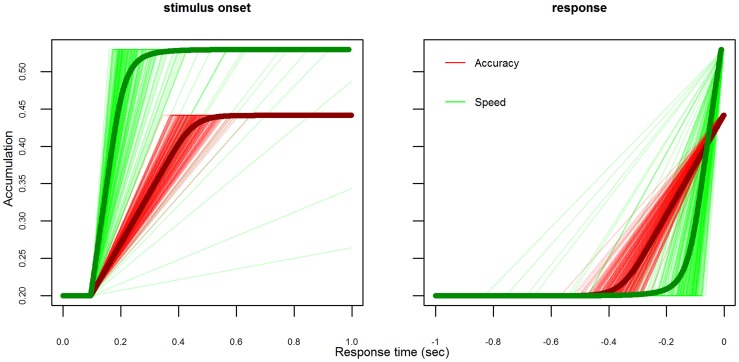
Sample accumulation trajectories, for monkey Q, for the accumulator corresponding to a neuron with a target in its receptive field. To correspond with neural data, only trajectories corresponding to correct decisions are displayed. Mean trajectories overlaid as heavy lines. Left panel displays paths aligned on stimulus onset. Right panel displays paths aligned on response. Only speed and accuracy emphasis conditions plotted due to high similarity between neutral and speed emphasis.

A strength of Heitz and Schall's [26] iA model was that its predicted evidence accumulation trajectories were qualitatively similar to the observed firing rate trajectories of movement neurons from the FEF. This was also true of our LBA model. Figure 3 shows the evidence accumulation trajectories predicted by the LBA model, for monkey Q (trajectories for monkey S are shown in Figure S4). Whether the sample trajectories from the LBA model are aligned on stimulus onset (left panel) or on response (right panel), the qualitative patterns are broadly consistent with with the neural data and those predicted by Heitz and Schall's iA model (their Figure 6B). However, one important discrepancy between the LBA model's predicted sample paths and the neural trajectories regards the time taken for stimulus registration (i.e., perception). Heitz and Schall's Figure 2C shows that neural firing rates begin to discriminate between target and distractor stimuli around 150 msec. after stimulus onset, but that the precise timing of this discrimination differs between the speed, neutral and accuracy emphasis conditions (by about 19 msec.). Our LBA model as described so far does not include any mechanism to account for these differences. However, it could easily do so in the same manner as Heitz and Schall's iA model; that is, by allowing the flexibility to estimate different parameters for the non-decision time (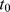) in the three conditions.

## Discussion

### Limitations and future directions

Heitz and Schall [26] (see also [35]) concluded that there was a disconnect between cognitive and neural accounts of evidence accumulation. This conclusion was based on showing that a particular standard cognitive evidence accumulation model fit the behavioral data, but not the neural data. Instead, we have shown that a standard cognitive model can fit both the behavioral and neural data recorded by Heitz and Schall. This is not to suggest that the model we have used is the best or most complete account of the data. Both the behavioral and neural data could certainly be modelled in greater detail. For example, the monkeys' task was not a two-choice decision, as assumed by by Heitz and Schall (an assumption we also adopted to make our modelling efforts comparable). The task was really a choice between eight options, which should more accurately be modelled by a race between eight accumulators. Such a race is not equivalent, under any parameter settings, to a race between two accumulators.

Another aspect of the data that deserve further attention is the nature of responding in the accuracy-emphasis condition. We made the simple assumption that those responses were a mixture of careful decisions along with some decisions that were identical to responses made under speed emphasis. This assumption is probably too simple, and deserves further investigation. Very fine-grained analysis of the response time distributions (see Figure S5) highlights the extra detail that might be modelled in these data. There are some signs in Figure S5 that the monkeys might not have been treating the accuracy-emphasis condition as a regular choice task at all. Rather – and in agreement with the reward procedures imposed on the monkeys – they might have been treating this condition as a “response signal” task, withholding their responses for some time after making a decision, in order to maximise their rewards. Most telling, the response time distributions in the accuracy condition are close to symmetric and almost Gaussian across a substantial range (see Gaussian curves on Figure S5). Gaussian distributions are never observed for response times from regular decision-making experiments, but they are observed in response signal experiments [36]. Further, Heitz and Schall's Figure 4C suggests that neural activity might not have been the same in those trials from the accuracy-emphasis condition where the monkeys neglected the cue as in some trials from the speed-emphasis condition. This suggests that our simple assumption that cue-neglect trials were identical to regular speed-emphasis trials might be too simple. Perhaps cue neglect trials resulted in a speed-accuracy setting somewhere between speed-emphasis and accuracy-emphasis conditions. These questions are beyond the scope of the current paper, but deserve further investigation, ideally with a complete model that is simultaneously applied to the behavioral and neural data [25].

An important advantage of Heitz and Schall's iA model is its ability to account for neural data downstream from the cortical recordings. Heitz and Schall's cortical recordings were from the frontal eye fields, using neurons previously identified as instantiating the process of evidence accumulation. These neurons subsequently influence neurons in the brain stem which are closely tied to response execution (generating eye movements, in this experiment). Different from the cortical neurons, these brain stem neurons have very constant firing rate thresholds between speed-emphasis and accuracy-emphasis conditions. If the leaky integrator component of Heitz and Schall's iA model is identified with these brain stem neurons, and if the decision threshold is assumed to occur in the brain stem, not the cortex, then the iA model neatly accounts for the constant (across emphasis conditions) firing rate thresholds observed in the brain stem as well as the varying firing rate thresholds observed in the cortex. This account remains to be carefully tested, however. For example, an assumption that the decision threshold resides in the brain stem, and that the threshold never changes, makes the strong prediction that *all* speed-accuracy tradeoffs must be accomplished by parameter settings similar to those observed here: higher thresholds and drift rates for speed than accuracy conditions. This is certainly not the case in many analyses of behavioral data. Further, if the decision threshold really does reside in brain stem neurons related to eye movements, this leaves open many questions about the effect of changing modality. For example, if the decisions were made without any reference to the visual system (such as finger-button responses to audio stimuli) would the eye-movement neurons still control the decision threshold? Such questions require further investigation.

## Summary

Heitz and Schall [26] report important new data – direct recordings from neurons implicated in decision making in monkeys, while the monkeys adjust their speed-accuracy tradeoff. When Heitz and Schall fit an overly-restricted version of a conventional decision-making model to the behavioral data alone, ignoring the neural constraints, the predictions of the behavioral model were inconsistent with the neural data. This prompted the addition of a new mechanism to the decision-making model with more complicated dynamics for the accumulation of evidence. The development of a new theory for simple perceptual decision-making is no small thing. In the interests of cumulative science, new theories are evaluated against many benchmarks that have been uncovered by the half-century of behavioral investigations of decision-making [9], [37], [38]. The new iA model developed by Heitz and Schall has not been subjected to these tests. In addition, the iA model does not permit practical mathematical solutions, which limits its application.

In contrast, we have shown that a conventional and well-validated decision-making model is able to account for the key patterns in both the behavioral and neural data recorded by Heitz and Schall. The detailed structure of our model's parameter settings was inspired by the neural activity, and also agrees with the parameter constraints used for Heitz and Schall's new iA model. The parameter estimates from our model accurately reflect important patterns in the neural activity data, and the predictions from our model match the fine-grained behavioral data, including full response-time distributions.

## Conclusions

Our conventional accumulator model accommodated both the behavioral and neural data reported by Heitz and Schall [26]. This calls into question the justification for rejecting a simple link between conventional cognitive and neural accounts of decision-making. The reason our LBA-based analysis reached this integrative conclusion while Heitz and Schall's did not is due to differences in the underlying logic. Heitz and Schall did not allow their initial LBA model analyses to be informed by the neural data at all. Rather, they analysed the behavioral data in isolation and then compared the resulting model predictions to the neural data. This strict approach does not align with the scientific goal of identifying models which are able to simultaneously account for both neural and behavioral data.

Heitz and Schall's detailed recordings from individual neurons engaged in decision making could never be obtained from human participants. Our analyses showed that a cognitive theory which standardly accounts for human data can also account for these data from monkeys, confirming again that primate experiments are valuable for understanding human cognition. However, our analyses also suggest that care needs to be exercised in extrapolating between species, and also across the different experimental procedures used for monkeys and people. For example, the monkeys in Heitz and Schall's experiments appeared to have default speed-accuracy settings that strongly emphasised speed over accuracy: data from the “neutral” condition, in which monkeys were free to choose their own speed-accuracy setting, were quite similar to data from the speed-emphasis condition, but very different from the accuracy-emphasis condition. This default setting is the opposite of that generally observed in humans, where behavior in a neutral condition is usually very similar to behavior under accuracy emphasis [17], [39]. It is an open question whether humans might behave similarly to monkeys when tested under identical procedures.

## Supporting Information

Figure S1 Markov chain Monte Carlo sampling chains for each parameter, for monkey Q and S. Only burnt in samples are displayed (10000–15000 iterations).
(TIF)

Figure S2 Marginal posterior distributions over each parameter for monkey Q and S.
(TIF)

Figure S3 Resultant fit for an over–constrained version of the model variant in the main text. Probability distribution functions over response time and choice. Observed data, plotted as coloured histograms. Model posterior prediction distributions are overlaid as lines.
(TIF)

Figure S4 Sample accumulation trajectories, for monkey S, for the accumulator corresponding to a neuron with a target in its receptive field. To correspond with neural data, only trajectories corresponding to correct decisions are displayed. Mean trajectories overlaid as heavy lines. Left panel displays paths aligned on stimulus onset. Right panel displays paths aligned on response.
(TIF)

Figure S5 Fine grain structure of response time distributions displyed in Figure 1. Lines overlaid on the accuracy RT distributions represent Gaussian curves.
(TIF)

Text S1 Details of the sampling method via Markov Chain Monte Carlo integration with proposals generated via differential evolution.
(PDF)

Text S2 Details of over-constrained version of the model variant in the main text.
(PDF)
