# Supplementary figures and images for: Brain and Behavior in Decision-Making
Source: PLoS Comput Biol. 2014 Jul 3;10(7):e1003700. doi: 10.1371/journal.pcbi.1003700 (PMC4081035; doi:10.1371/journal.pcbi.1003700)

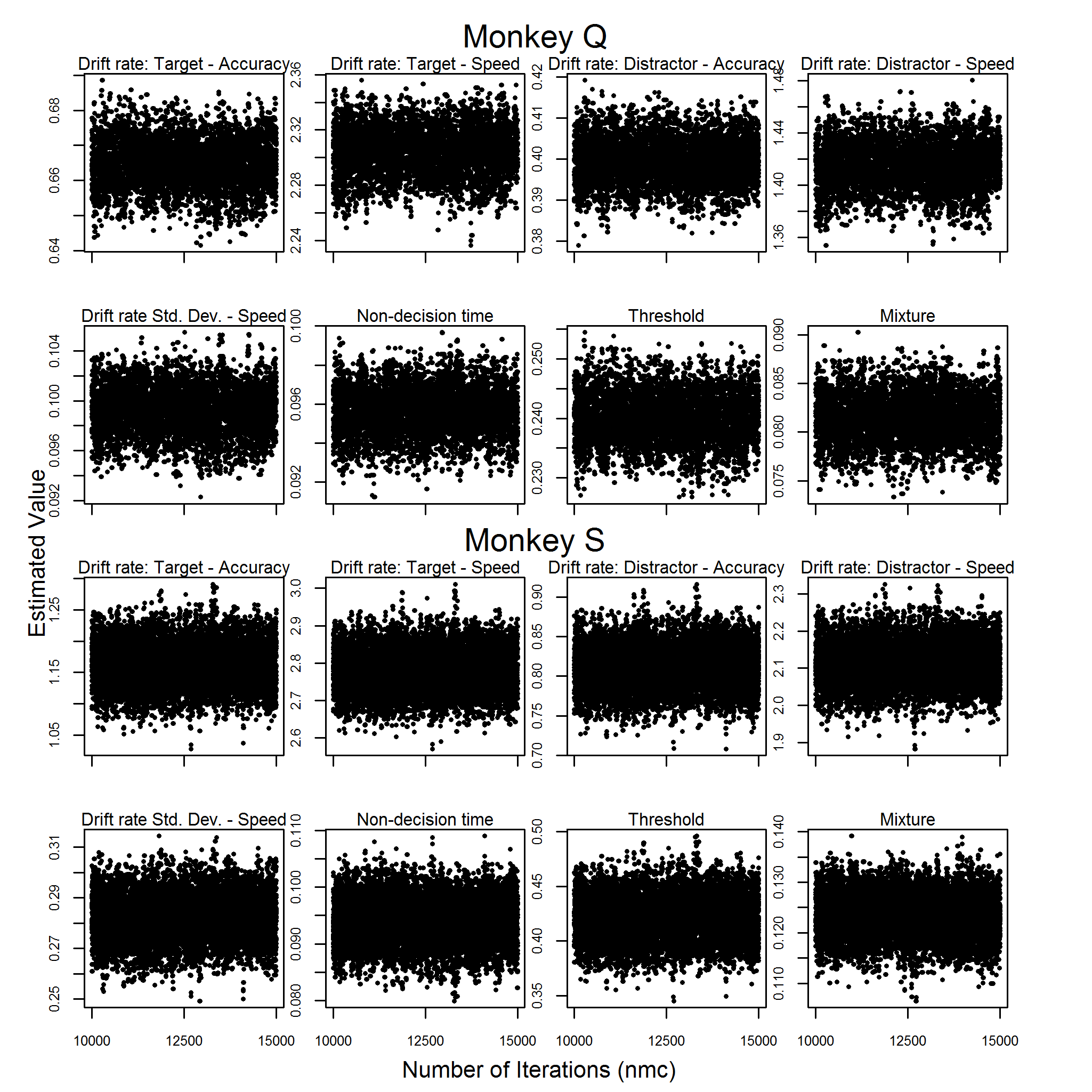

Supplement: Figure S1 — Markov chain Monte Carlo sampling chains for each parameter, for monkey Q and S. Only burnt in samples are displayed (10000–15000 iterations). (TIF) [file pcbi.1003700.s001.tif]

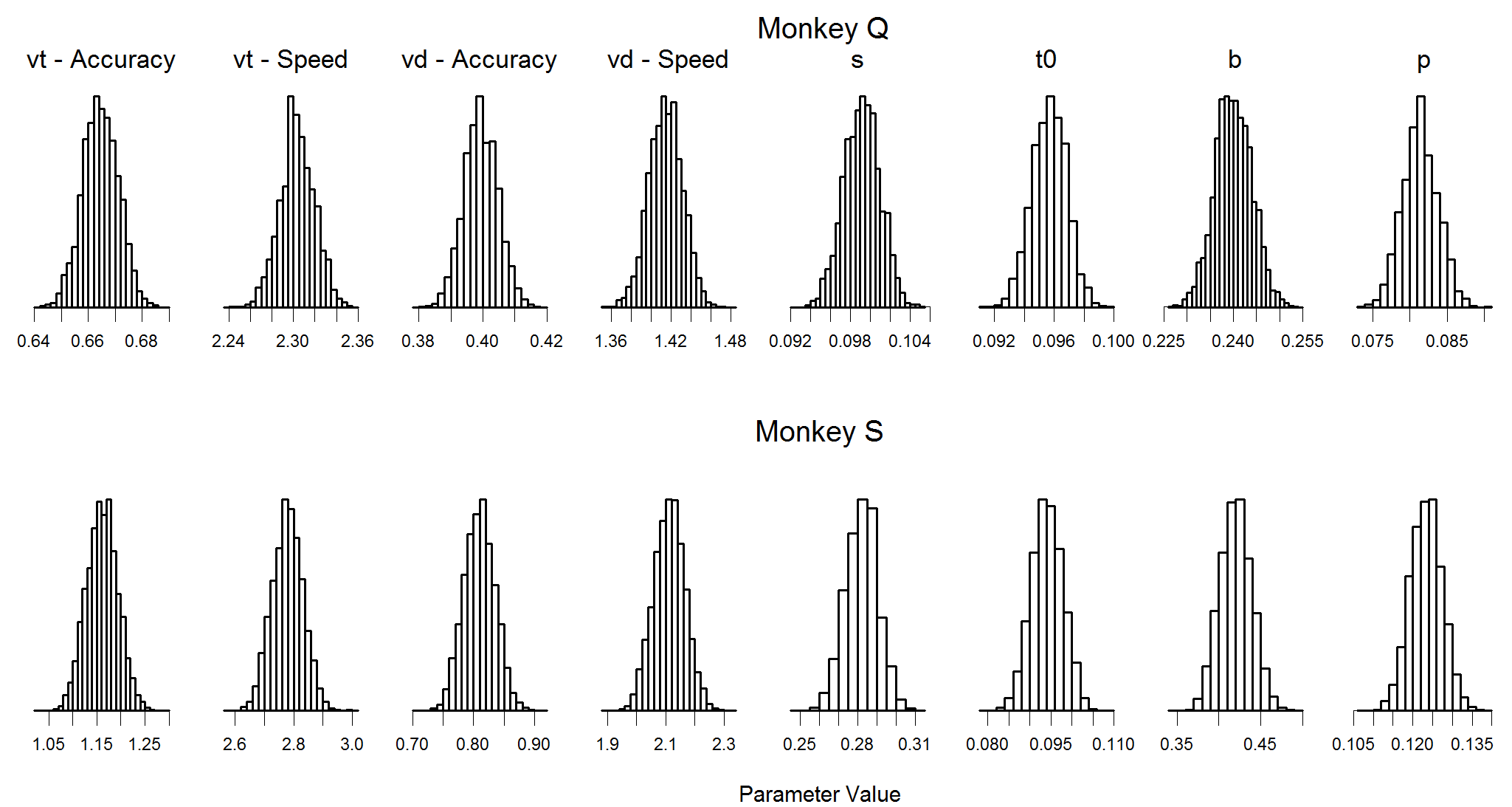

Supplement: Figure S2 — Marginal posterior distributions over each parameter for monkey Q and S. (TIF) [file pcbi.1003700.s002.tif]

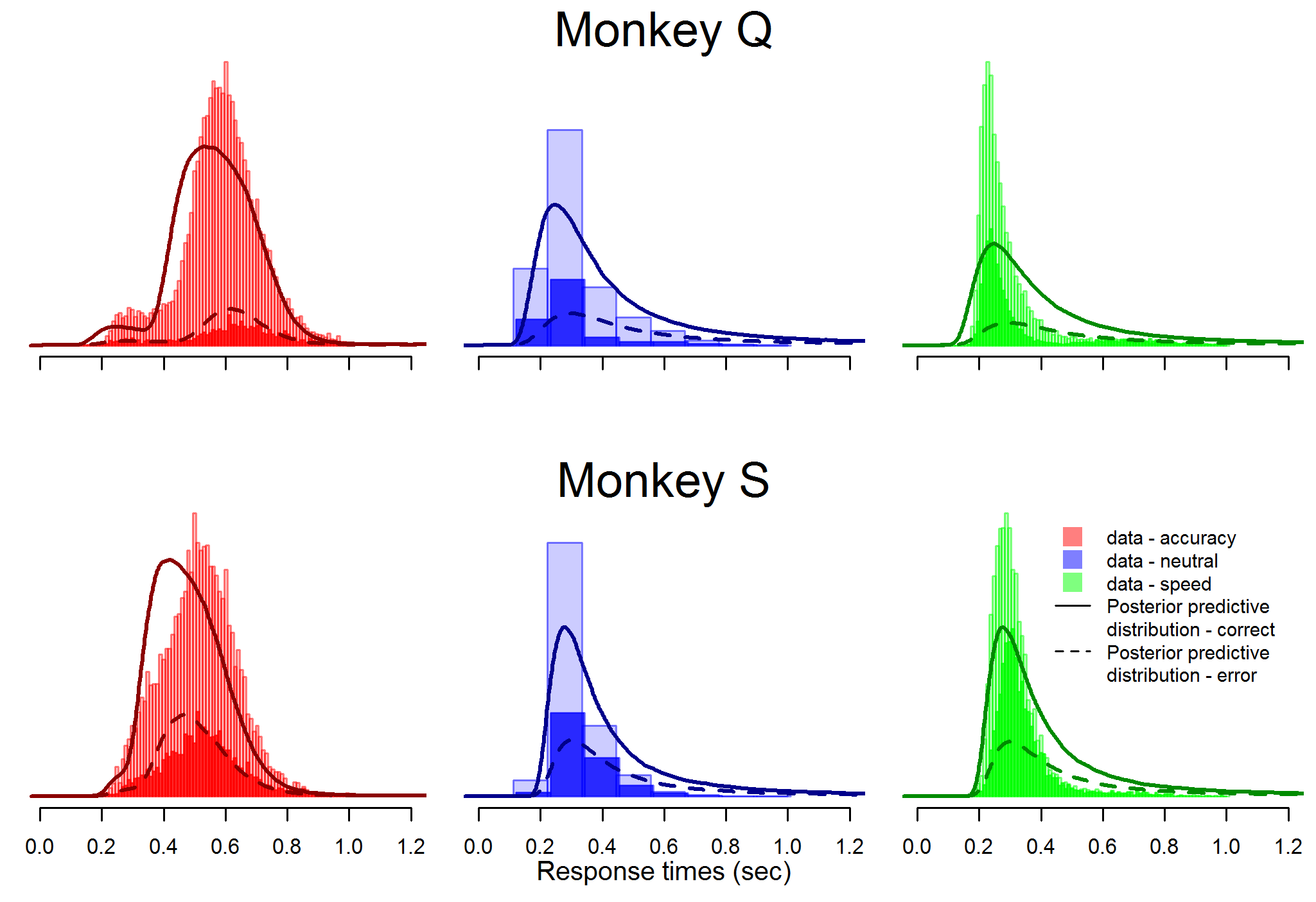

Supplement: Figure S3 — Resultant fit for an over–constrained version of the model variant in the main text. Probability distribution functions over response time and choice. Observed data, plotted as coloured histograms. Model posterior prediction distributions are overlaid as lines. (TIF) [file pcbi.1003700.s003.tif]

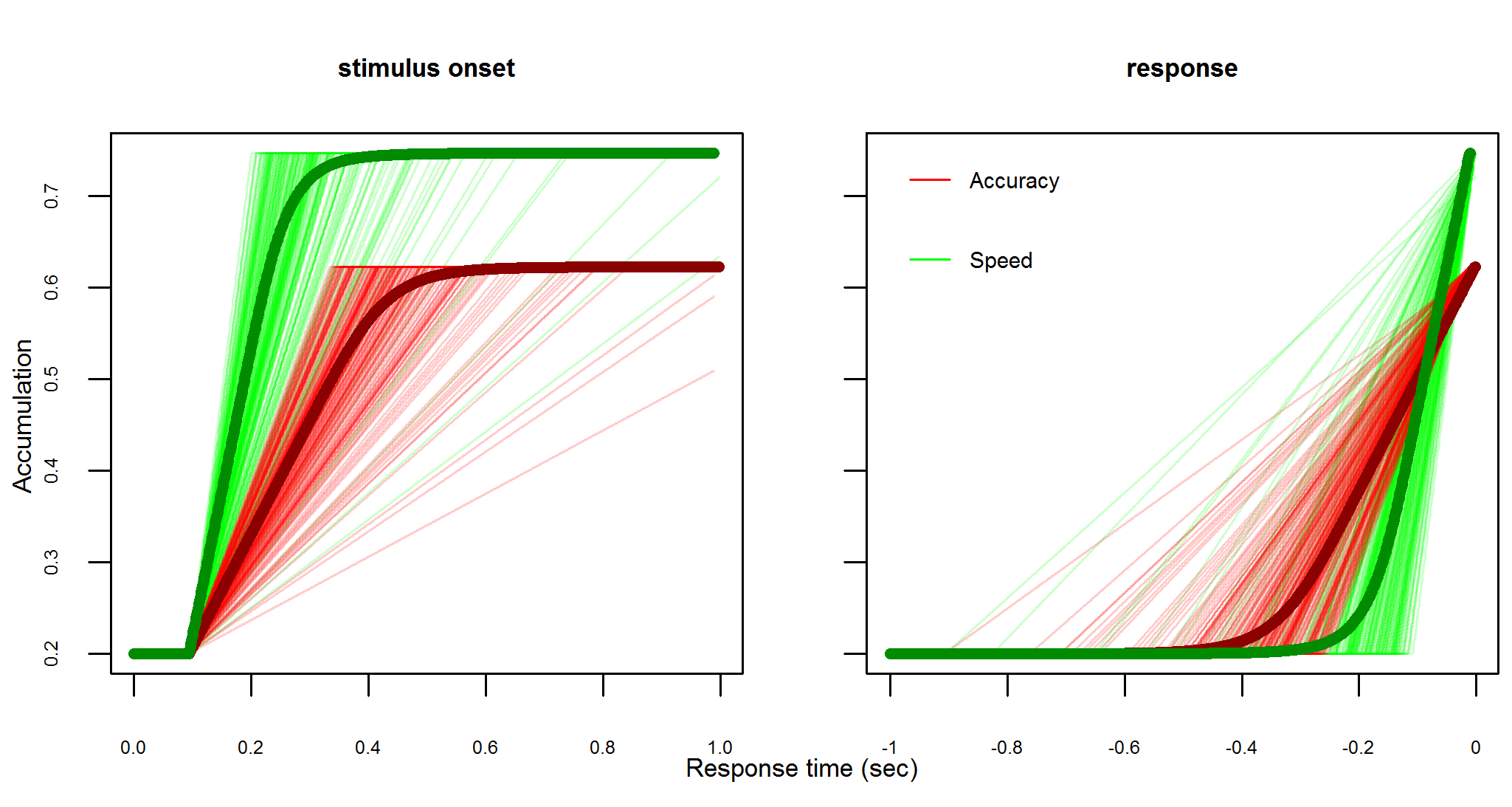

Supplement: Figure S4 — Sample accumulation trajectories, for monkey S, for the accumulator corresponding to a neuron with a target in its receptive field. To correspond with neural data, only trajectories corresponding to correct decisions are displayed. Mean trajectories overlaid as heavy lines. Left panel displays paths aligned on stimulus onset. Right panel displays paths aligned on response. (TIF) [file pcbi.1003700.s004.tif]

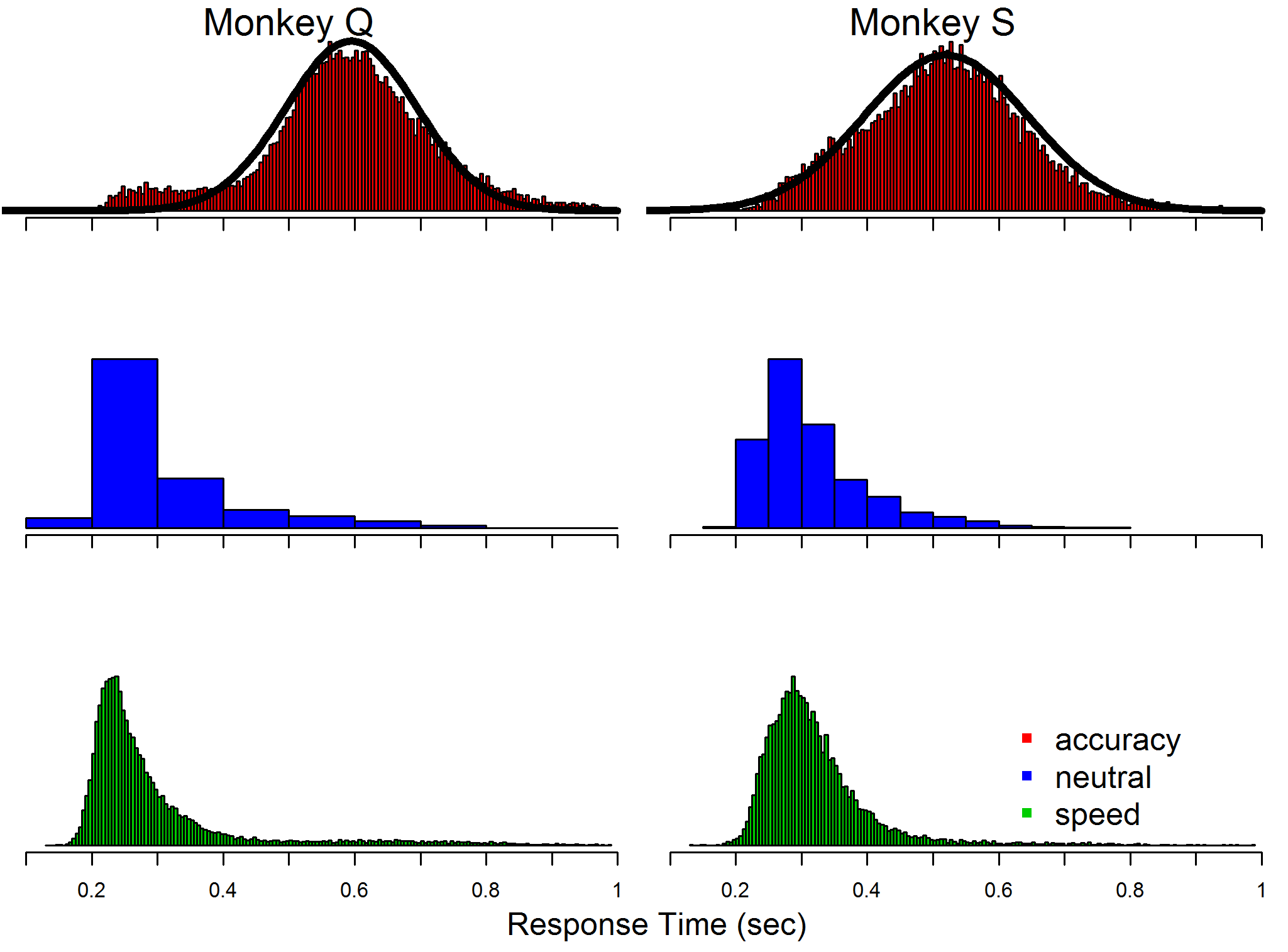

Supplement: Figure S5 — Fine grain structure of response time distributions displyed in Figure 1. Lines overlaid on the accuracy RT distributions represent Gaussian curves. (TIF) [file pcbi.1003700.s005.tif]
