# Supplementary material for: Brain and Behavior in Decision-Making
Source: PLoS Comput Biol. 2014 Jul 3;10(7):e1003700. doi: 10.1371/journal.pcbi.1003700 (PMC4081035; doi:10.1371/journal.pcbi.1003700)
Supplement: Text S1 — Details of the sampling method via Markov Chain Monte Carlo integration with proposals generated via differential evolution. (PDF) [file pcbi.1003700.s006.pdf]

Sampling Procedure

Prior Distributions

We used broad prior distributions, and their influence was especially small because of the very large sample of data recorded from each monkey (around 18,000 decisions each). The prior for the mixture parameter was the beta distribution $\beta(1,8)$. We used this skewed distribution because of our expectation that the monkeys should mostly follow the experimenters' instructions, with relatively infrequent lapses (most of the mass of this distribution is below 0.3). The prior distributions for all other parameters were normal distributions truncated to positive values, with parameters:

- drift rate mean parameters: $\mu = 2, \sigma = 2$
- drift rate standard deviation parameters: $\mu = 1, \sigma = 1$
- non-decision time $\mu = 0.15, \sigma = 0.15$
- decision threshold: $\mu = 1, \sigma = 1$

Initialising the chains

Chains were initialised using random draws from distributions similar to the prior distributions. Importantly, exactly the same distributions were used for speed emphasis and accuracy emphasis parameters, ensuring any difference in posterior distributions over these parameters was due to the data. Truncated normal distributions – positive only – were used to sample the initial points for target drift rates for speed and accuracy (μ=4, σ=1); distractor drift rates for speed and accuracy conditions (μ=3, σ=1); standard deviation of the drift rates for speed condition (μ=1, σ=0.2) and; response threshold (μ=1.5, σ=0.3). Initialization points for non-decision time were sampled uniformly between zero and 0.15sec. Initialization points for the mixture parameter were sampled beta distribution (α=1, β=6).

Sampling details

We drew samples from the posterior distributions over parameters using Markov Chain Monte Carlo with proposals generated by differential evolution [1]. Differential evolution relies on two tuning parameters, and we used conventional settings for each, following choices reported by [1]. The step size scaling parameter was determined by the number of free parameters ($K = 8$), namely: $\gamma = \frac{2.38}{\sqrt{2K}} = 0.595$. The perturbation term was set at a value much smaller than all estimated parameters: $b = 0.001$.

We drew 15,000 samples from each of 20 Markov chains. We discarded the first 10,000 samples as burn-in. We discarded two "stuck" chains for monkey S and four for monkey Q. Those chains were associated with markedly lower likelihood than all other chains, even after burn-in (see Figure S1 for all sampling chains for both monkeys). Marginal posterior distributions are shown in Figure S2.

References

1. Turner, B. M., Sederberg, P. B., Brown, S. D. and Steyvers, M. (2013) A method for efficiently sampling from distributions with correlated dimensions. Psychological Methods 18: 368–384.
