# Supplementary material for: Brain and Behavior in Decision-Making
Source: PLoS Comput Biol. 2014 Jul 3;10(7):e1003700. doi: 10.1371/journal.pcbi.1003700 (PMC4081035; doi:10.1371/journal.pcbi.1003700)
Supplement: Text S2 — Details of over-constrained version of the model variant in the main text. (PDF) [file pcbi.1003700.s007.pdf]

Enforcing Over–Constraint on a Model

As outlined in the main text, Heitz and Schall [1] over–constrained their LBA model. We enforced the same parameter constraints as Heitz and Schall, in that we fixed the variance of the distractor drift rate at 1 and constrained the sum of target and distractor drift rates at 1. Figure S3 displays the effect of this over–constraint on the model variant described in the main text. The fit has been substaintially hampered. We calculated fit statistics using DIC [2]. For monkey Q, minimal constraint model varaint: DIC = -127239.9, over–constrained model variant: DIC = -5773.8. For monkey S, minimal constraint model varaint: DIC = -41144.3, over–constrained model variant: DIC = -17682.3. Smaller DIC values are preferred.

References

1. Heitz, R. P. and Schall, J. D. (2012) Neural mechanisms of speed-accuracy tradeoff. Neuron 76: 616–628.

2. Spiegelhalter, D. J., Best, N. G., Carlin, B. P. and van der Linde, A. (2002) Bayesian measures of model complexity and fit. Journal of the Royal Statistical Society B 64: 583–639.
